# Patient relevant outcome 7 years after total hip replacement for OA - a prospective study

**DOI:** 10.1186/1471-2474-11-47

**Published:** 2010-03-11

**Authors:** Anna-K Nilsdotter, Fredrik Isaksson

**Affiliations:** 1 Department Orthopedics, Lund University, Lund, Sweden; 2 Department Orthopedics, Halmstad Central Hospital, Halmstad, Sweden

## Abstract

**Background:**

To investigate prospectively the patient-relevant outcome 7 years after total hip replacement (THR) for osteoarthritis (OA).

**Methods:**

219 consecutive patients (120 women) with primary OA, mean age 71 (range 50-92) were assigned for THR. They were examined preoperatively, at 3, 6, 12 months, and at 4, 5 and 7 years postoperatively with the self-administered questionnaires SF-36 and WOMAC. Supplementary questions regarding postoperative complications, general co-morbidity, social circumstances and patient satisfaction were asked at the three last follow-ups. A reference group, 117 subjects (67 women), mean age 72 (range 52-92) without hip complaints were recruited from the community and investigated at the same times.

**Results:**

151/170 (89%) of the patients and 65/74 (88%) of the reference group participated at the 7 year follow-up. The best postoperative result was reported one year postoperatively. At the 7 year follow up there was a significant difference between the patients and controls in SF-36 physical function (PF) and role physical (RP) but not of WOMAC function. There was no difference in frequency of co-morbid conditions between those operated and the reference group, but those operated were in greater need of walking aid (46% vs. 8% p < 0.0001) and reported more regional and widespread pain (68% vs. 53% p < 0.05).

**Conclusion:**

This study shows that in an unselected cohort the patients experience a similar health-related quality of life as a reference group of a similar age and sex structure 7 years after THR except for general physical function where the patients score worse.

## Background

Total hip replacement (THR) is one of the most common interventions in orthopedic surgery. A continued increase of incidence appears likely with a growing elderly population who expects to live an active life. In a review in Lancet 2007 [1]THR was identified as the operation of the century since THR has revolutionized the management of elderly patients with osteoarthritis (OA) with very good long term results in terms of prosthetic survival and cost-effectiveness.

In patients suffering from severe osteoarthritis (OA) total joint replacement is known to be the most effective treatment [2]and it offers the patient pain relief as well as improved physical function [3,4]. The main indication for THR is severe pain [5]. However, patients' expectations after THR have changed [6]. Today, many patients participate in broad range of physical demanding activities and expect to live an active life in the future. Within orthopedic surgery the assessment of effectiveness has traditionally focused on the surgical and technical aspects. Recently measurement of patient reported health outcomes has provided an alternative source of valuable information [7]. Numerous follow-up studies after THR have been performed over the last decade with a follow up time of one to two years with patient-reported outcomes [6,8-13]. Information on patient reported long term results is thus limited whereas there are numerous papers concerning long-term prosthetic survival.

The main purpose of this study was to investigate prospectively the patient-relevant outcome seven years after THR for OA with a focus on pain and physical function.

## Methods

### Patients

Two hundred and nineteen patients (120 women, 55%), mean age 71 years (range 50-92) with primary OA were consecutively included in the study. All patients had a unilateral THR performed at the department of Orthopedics at Halmstad Central Hospital between September 1995 and October 1998 [4].

### Reference group

For each patient, that was included during September 1997 to October 1998, 3 subjects were identified in the National Population records. That resulted in 258 subjects. The subjects were matched to the patients by age, sex and municipality. The questionnaires were sent to these 258 subjects with an explanatory cover letter. In the cover letter they were told not to respond if they had hip complaints. Hip complaints were defined as pain or diminished range of motion in their hips.

One hundred and seventeen individuals (45%) answered the first inquiry. Their mean age was 72 (range 52-92, 57% women, 43% men) [4]. One hundred and forty one subjects did not answer the first inquiry. Their mean age was 72 years (range 50-90, 52% women, 48% men). No reminder letters were sent as the number was regarded as sufficient for comparison of groups. Additional letters with the same questionnaires were sent to these subjects at the same intervals as for the patients.

### Design of the study

Self report with the patient administered questionnaires SF-36 and WOMAC was obtained preoperatively, at 3, 6, 12 months and after 4, 5 and 7 years postoperatively. At the last three follow-ups supplementary questions were asked.

### Questionnaires

*SF-36 *is a widely used generic outcome measure [14] and it consists of 8 domains; PH (physical function), RP (role-physical), BP (bodily pain), GH (general health), VT (vitality), SF (social functioning), RE (role-emotional) and MH (mental health). The SF-36 is self-explanatory and takes about 10 minutes to complete. The SF-36 is scored from 0-100, 0 indicating extreme problems and 100 indicating no problems. The Acute Swedish version of the SF-36 was used [15].

*WOMAC *(Western Ontario and McMaster Universities Osteoarthritis Index) was used as the disease specific outcome measurement (LK 3.0). WOMAC is well known and a self administered instrument validated for OA in the lower extremities and for evaluating outcome after THR [16]. It consists of 24 items grouped in to three categories: pain (five questions), stiffness (two questions) and physical function (seventeen questions). It is reliable and valid for Swedish conditions [17]. To enhance the interpretation WOMAC is transformed to a 0-100 worst to best scale [17-19]. Because this instrument was not available and validated for Swedish conditions when the study was started, it was used for the last 92 patients included.

### Additional questions

Questions concerning postoperative complications, preoperative and postoperative co-morbidity, social circumstances and patient satisfaction were asked at the 4, 5 and 7 year follow-up. The patients and the reference group received the same questions except those relating to postoperative complications.

#### Postoperative complications

Three questions dealt with serious postoperative complications, dislocation of the prosthesis, deep infection in the hip joint and reoperation. The definition of postoperative complication in this study referred to a positive answer in one of these three questions.

#### General co-morbidity

Fourteen questions were asked about intercurrent diseases preoperatively and in the present situation [20,21]. Questions were asked about the presence of 10 comorbid conditions or body areas with problems (heart, hypertension, peripheral arteries, lung, diabetes, neurological problems, cancer, ulcer, kidney disease, vision).

#### Musculoskeletal co-morbidity

Two questions were asked about the need of walking assistance and walking distance preoperatively and in the present situation [22,23], two questions were asked about the need for analgesics due to pain in the operated hip or due to pain elsewhere. One question was asked about the experience of regional or widespread pain lasting more than three months during the past 12 months [20]. One question was asked about joint replacement in the contralateral hip or in the knees since the THR. The final question concerned fractures in the spine, wrist, hip or elsewhere.

#### Social circumstances

One question addressed living circumstances preoperatively and in the present situation. One question was asked about the civil status and one about the main profession and the present profession or occupation.

#### Patient satisfaction

One question dealt with patient satisfaction after surgery:" Overall, how satisfied are you with the result of your hip replacement surgery". The alternative answers were: very satisfied, satisfied, dissatisfied, very dissatisfied.

### Statistics

Statistical analysis was performed using SPSS 17.0. For comparison of preoperative and postoperative questionnaire data for the patients the Wilcoxon's signed rank test was used. Multiple logistic regression analysis was taken in account to comparison between patients and controls. Age, sex and SF-36 subscale were independent variables and patient and controls dependent variables. The municipality variable was not included as covariate in the analyses since the controls were stratified after belongings to municipality. Chi-square test was used when comparing the patients and the controls using walking aids, walking distance, number of co-morbidities, low back pain, and widespread pain. Significance level was set at 0.05.

## Results

Of the 219 patients, 21 died during the 7 year follow up period and 47 did not participate. Thus the result of 151 patients (83 women, 55%), with a mean age at surgery of 70 years (range 50-88) are presented for a follow-up time of 7 years (Table 1). Of the 117 subjects in the reference group, 11 died during the follow-up period and 41 declined participation. Thus the results for 65 (39 women, 60%) subjects, with a mean age at the start of the study of 70 years (range 52-83) are presented (Table 1).

**Table 1:** Patients and controls during the study.

Follow-up time	baseline	4 years	5 years	7 years	
**Patients**	219	198	170	151	
Dead		8	13		21
Missing*		13	15	19	47
**Controls**	117	83	77	65	
Dead		8		3	11
Missing*		26	6	9	41

*Missing due to dementia, unknown address or declined to participate.

### Postoperative self-reported results

#### SF-36

The patients improved significantly in all subscales of SF-36 between base line and 12 months postoperatively. Between 12 months and 7 years postoperatively there was a significant decline in all subscales except RE (role emotional) (Table 2).

**Table 2:** Preoperative, 12 months and 7 years postoperative mean scores and (standard deviations) for the SF-36 subscales, for the patients, mean age 70 (50-88).

	Patients preoperative (n = 151)	Patients 12 months postoperative (n = 151)	p-value preop-12 months	Patients 7 years Postoperative (n = 151)	p-value 12 months-7 years
SF-36 PF	31 (19.4)	68 (21.1)	<0.001	54 (27.2)	<0.001
SF-36 RP	9 (21.1)	61 (41.2)	<0.001	45 (44.6)	0.001
SF-36 BP	31 (15.8)	75 (22.7)	<0.001	63 (28.1)	<0.001
SF-36 GH	68 (19.8)	73 (21.6)	0.008	63 (22.4)	<0.001
SF-36 VT	49 (20.2)	73.4 (20.6)	<0.001	59 (46.4)	0.003
SF-36 SF	63 (26.4)	88 (21.3)	<0.001	62 (23.8)	<0.001
SF-36 RE	37 (43.5)	74 (36.8)	<0.001	81 (23.2)	0.10
SF-36 MH	70 (21.2)	83 (18.9)	<0.001	79 (19.1)	0.03

The scale is 0-100, worst to best. The p-value is calculated between preoperative outcome and 12 months postoperative outcome and between 12 months postoperative outcome and 7 years postoperative outcome.

#### WOMAC

There was a significant improvement in all three subscales of WOMAC between baseline and 12 months postoperatively [4]. At the final follow-up there was no significant decline in the WOMAC subscales compared to 12 months (Table 3).

**Table 3:** Preoperative, 12 months and 7 years postoperative mean scores and (standard deviations) for the three WOMAC subscales, for the patients, mean age 70 (50-88).

Subscale	Patients preoperative (n = 75)	Patients 12 months postoperative	p-value	Patients 7 years postoperative	p-value
WOMAC pain	44 (16.5)	85 (16.4)	<0.001	86 (16.5)	0.69
WOMAC stiffness	38 (15.9)	77 (18.7)	<0.001	78 (22.1)	0.63.
WOMAC function	38 (14.8)	79 (16.7)	<0.001	76 (21.1)	0.12

The scale is 0-100, worst to best. The p-value is calculated between preoperative outcome and 12 months postoperative outcome and between 12 months postoperative outcome and 7 years postoperative outcome

#### Postoperative complications

At seven years after surgery complications were reported from 5 patients (3%). 4 patients suffered from a deep infection and 2 of them were reoperated. 1 patient had a dislocation of the prosthesis.

#### General comorbidity

19% of the patients reported 2 or more co-morbidities at the 7 year follow up (Table 4).

**Table 4:** Co-morbidities reported 7 years after THR.

	Patients N = 151	Controls N = 65	P value
Walking aids	46%	8%	<0.001
Walking ability >3 km	59%	70%	0.15
Co-morbidities ≥ 2	19%	31%	0.08
Low back pain	29%	19%	0.17
Wide spread pain	15%	8%	0.05
Regional pain	53%	45%	0.05
Unilateral hip pain	20%	10%	0.07

The most common co-morbidity was hypertension which 26% of the patients suffered from.

#### Musculoskeletal comorbidity

Almost half of the patients were in need of walking aids at the last follow-up, though the majority reported a walking distance more than 3 km. One third of the patients suffered from low back pain and as many as half of them from regional pain (Table 4).

#### Social circumstances

40% of the patients lived alone at the last follow-up. 54% of the patients were homeowners and 45% lived in an apartment.

### Comparison between patients and controls

At the final follow-up there was a significant difference, when adjusted for age and sex, between the patients and the reference group in the subscale PF (physical function), VT (vitality) and RP (role physical) where the patients scored worse (Table 5).

**Table 5:** Final follow up (7 years) postoperative mean scores and (standard deviations) of the SF-36 and WOMAC subscales, for the patients, mean age 70 (50-88), and the reference group, mean age 70 (50-83)

Subscale	Patients 7 years postoperative (n = 151)	Reference group 7 years Postoperative (n = 65)	p-value
SF-36 PF	54 (27.2)	69 (31.3)	0.01
SF-36 RP	45 (44.6)	60 (46.0)	0.05
SF-36 BP	63 (28.1)	69 (26.9)	0.19
SF-36 GH	63 (22.4)	62 (25.0)	0.94
SF-36 VT	59 (46.4)	72 (43.0)	0.05
SF-36 SF	62 (23.8)	65 (21.8)	0.36
SF-36 RE	81 (23.2)	79 (24.7)	0.53
SF-36 MH	79 (19.1)	72 (43.0)	0.90
WOMAC pain	86 (16.5)	91 (18.2)	0.05
WOMAC stiffness	78 (22.1)	89 (20.0)	<0.001
WOMAC function	76 (21.1)	73 (23.8)	0.56

The p-value is calculated using a multiple logistic regression analysis, adjusted for age and sex.

At that time there was no difference between the patients and the reference group in WOMAC physical function but the patients reported more pain and more stiffness than the reference group (Table 5).

Almost half of the patients were in need of walking aid at the last follow-up compared to the reference group where only 8% had those needs. However there was no difference in walking ability between the two groups (Table 4). There was no significant difference in the presence of low-back pain but a difference in widespread pain or regional pain between patients and controls (Table 4).

### Outcome in relation to preoperative pain and function

**Figure 1:**
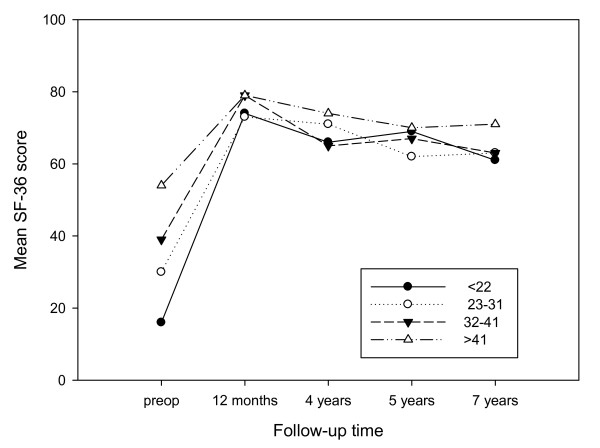
**SF-36 pain scores before and after THR**. The mean SF-36 pain scores at the preoperative, 12 months, 4 year, 5 year and 7 years follow-up according to preoperative SF-36 quartiles.

To examine the possible influence of preoperative pain reported by SF-36 on postoperative SF-36 pain at 12 months, 4 years, 5 years and at the 7-year follow-up, the patients were analyzed according to preoperative SF-36 pain score quartiles (≤ 22, 23-31, 32-41, ≥ 41). The mean SF-36 pain score for each group at the different assessments are shown in Figure 1. At the 12 months follow-up the patients with a preoperative SF-36 pain score in the lowest preoperative quartile (≤ 22) reached almost the same level as the patients in the upper preoperative quartiles. At the final follow-up there was a difference between the lowest and highest quartile of 10 scores points (61 vs.71) (Figure 1).

**Figure 2:**
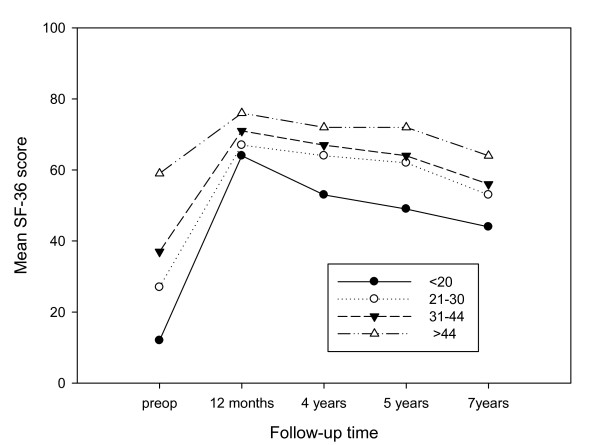
**SF-36 PF scores before and after TKR**. The mean SF-36 PF scores at the preoperative, 12 months, 4 year, 5 year and 7 years follow-up according to preoperative SF-36 quartiles.

The mean postoperative SF-36 PF score for the patients were stratified according to their preoperative SF-36 PF score quartiles (≤ 20, 21-30, 31-44, 44) (Figure 2). The patients in the lowest quartile had the greatest improvement in mean PF score at the 7-year follow-up, but was the group which declined the most between 12 months and 7 years (64-44. The difference between the patients in the lowest quartile and the highest at the final follow up was 20 score points (44 vs.64) (Figure 2).

### Satisfaction

96% of the patients were satisfied in general with the outcome at the seven year follow-up. As many as 95% of the patients were satisfied with their ability to practice leisure activities seven years after THR. 97% were satisfied with their pain relief and improved physical function.

## Discussion

Total hip replacement is a successful surgical intervention. This study confirms previous knowledge of improvement in physical function, reduced pain and improved health-related quality of life postoperatively [7]. Patients who receive a THR experience almost the same health related quality of life as a reference group of similar age and sex structure apart from physical function even seven years after surgery.

Most of the studies with patient reported data after total joint replacements have a follow-up period of 1-2 years [6,8,10-13,24]. The patient reported data at that time are generally good and coincide in many countries with the last clinical assessment after surgery. The present study is as far as we know one of few prospective long-term follow-up studies of THR with a focus on patient reported pain and physical function. In a previous study by Ng et al[25] they followed patients with various preoperative diagnosis (92.5% OA) 5 years after THR with SF-36 and concluded that the greatest improvement was seen 6 months after surgery.

Unfortunately they did not make a 1 year assessment of outcome but it would probably be in accord to our result. Furthermore, we had the possibility to follow a reference group during the whole follow-up period. The expected survival of the hip implant is at least 95% over 10 years http://www.jru.orthop.gu.se, hence, it would be interesting to know how the patients experience the results after such a long time. As far as we know there are no such data presented.

### Outcome in pain and physical function

The primary indication for THR is pain, although we know that the patients expect a high degree of improvement in physical function [6]. The present study shows very satisfying results concerning pain relief, even in the longer perspective, comparable with those of the matched reference group. This is in contrast to results after total knee replacement (TKR) where the patients report significantly more pain after five years [26]. However, that is based on one paper since there is still few long-term follow up studies after TKR. It is well established that gains in physical function after TKR and THR are delayed compared with pain relief [7]. After one year the patients report the same level of physical function as a matched reference group (data not shown) but at the coming follow-ups the patients decline significantly more than the reference group. This may be due to the placebo effect of surgery, since it has been shown in patients with OA that there is a significant placebo effect, especially for self-reported pain and function. The placebo effect is influenced by the strength of the active treatment [27]. Surgery and especially total joint replacements are considered to be a very strong treatment with effect sizes >0.8[28]. However, the decline in physical function over the years is more probably due to the musculoskeletal co-morbidity. In our study there is a significant difference in the presence of regional and widespread pain between patients and controls. The patients also reported more hip pain which could be due to a generalized OA which proceeded over years. The evidence of deteriorating physical function over years makes it important to focus on the postoperative rehabilitation [29,7]. Patients in the lowest quartile of physical function had inferior results over time compared with patients with a better baseline value. This is in accordance with previous studies where a worse preoperative physical function was shown to be a predictor of a worse postoperative physical function [4,30,31].

A similar analysis of pain relief related to their baseline values in SF-36 pain showed that the pain relief is excellent and independent of the baseline value.

It is interesting to notice in our study that the patients with the worst baseline score in SF-36 physical function made the relatively greatest improvement after one year. However, they are the same patients that make the largest decline to the last follow-up after seven years. It seems as if total hip replacement for patients with a very insufficient physical function only has a temporary effect. Nevertheless, one has to be aware of the risk that these results are an effect of the phenomenon of regression to the mean.

It has been shown in previous studies that disease severity varies greatly at time of surgery [32] and that there are large differences in preoperative pain and physical function between centers in different parts of the world [33]. The population that is described in our study seems to be representative since the patients' reports scores somewhere in the middle. However, it is still uncertain what effect it has on outcome.

### Patient reported outcome measures

In the present study we found significant differences in physical function compared to the reference group at the final follow-up by using SF-36 but not by using WOMAC. It seems probable that SF-36 has a better ability to capture the general musculoskeletal co-morbidity than WOMAC which focuses on the lower extremity.

### Satisfaction

Satisfaction is a complex phenomenon influenced by many factors but especially by expectations and outcome [34]. General satisfaction is, to our knowledge, even more complex and should not be used as primary outcome. It is too blunt of an instrument for that purpose. In the present study as many as 96% of the patients were satisfied with their THR as long as 7 years after the intervention. It is notable that the satisfaction concerning pain relief is comparable with the satisfaction of improved physical function (96-97%). The results are in contrast to a previous study of TKR where approximately 60% of the patients were satisfied with their pain relief after 5 years [26]. Thus, our study confirms what a number of previous studies have shown, that it is easier to get satisfied patients and a better outcome after THR than after TKR.

## Conclusion

Total hip replacement for osteoarthritis is a successful procedure. There is a marked change in most of the measures from pre to post surgery. This study shows that in an unselected cohort the patients experience a similar health-related quality of life as a reference group of a similar age, sex structure 7 years after THR except for physical function where the patients score worse. This may be explained by musculoskeletal co-morbidities such as progress in generalized OA.

## Competing interests

The authors declare that they have no competing interests.

## Authors' contributions

AKN designed the study, coordinated the data collection analyzed the data, performed the statistical analysis and drafted the manuscript. FI analyzed the data and helped to draft the manuscript. Both authors read and approved the final manuscript.

## Pre-publication history

The pre-publication history for this paper can be accessed here:

http://www.biomedcentral.com/1471-2474/11/47/prepub
